# International collaborative research and development (R&D) on traditional medicine and its contextual factors: a cross-sectional analysis from 1996 to 2022

**DOI:** 10.7189/jogh.16.04029

**Published:** 2026-02-06

**Authors:** Yinuo Sun, Jiyan Ma, Jingya Dong, Yiwu Gu, Myeong Soo Lee, Lin Ang, Yuming Liu, Yangmu Huang

**Affiliations:** 1 Department of Global Health, School of Public Health, Peking University, Beijing, China; 2 School of Public Administration, Southwestern University of Finance and Economics, Chengdu Sichuan, China; 3 School of Health Humanities, Peking University, Beijing, China; 4 KM Science Research Division, Korea Institute of Oriental Medicine, Daejeon, Republic of Korea

## Abstract

**Background:**

Traditional medicines can contribute to achieving universal health coverage, particularly in low- and middle-income countries where access to conventional treatments is limited. International collaboration is crucial to bridge the lag in modernised research and promote access to traditional medicines. This study focused on China’s global collaborative research and development (R&D) efforts on traditional medicine, in the hope of improving global recognition for traditional medicine.

**Methods:**

We conducted a cross-sectional study to analyse collaborative R&D outputs on Chinese patent medicines from 1996 to 2022. The study cohort included the collaborative outputs of scientific research, patent applications, and clinical trials between China and other countries. We analysed the outputs using data from the Web of Science, Worldwide Patent Statistical Database, and the International Clinical Trials Registry Platform. The Zero Inflated Negative Binomial regression model was employed to investigate the association between outputs and the characteristics of participating countries.

**Results:**

The majority of collaborative outputs (n = 964, 92.4%) originated from collaborations with high-income countries, with only 7.6% involving low- and middle- income countries (LMICs). The percentage of R&D collaborations with LMICs showed an increasing trend from 0% in 1996 to 11.7% in 2022. Most collaborations focused on non-communicable diseases (n = 912, 87.4%). Low-income countries accounted for a larger share of collaborative R&D on communicable diseases (14.3%) compared with high-income countries (1.4%). The total number of outputs was positively associated with the degree of cooperative institutionalisation and the collaborator’s traditional medicine development score.

**Conclusions:**

Gaps still remain in the involvement of low- and middle-income members compared with high income countries. Findings highlight the importance of encouraging greater engagement of low- and middle- income countries in global R & D collaboration on traditional medicine, particularly through South-South partnerships. Such collaborations should prioritise research agendas that address local health priorities, especially those related to communicable diseases.

Traditional medicine has long been regarded as an innovative health solution and has historically played a vital role in global health, especially in regions with limited access to conventional treatments [1,2]. There is increasing recognition that traditional medicine can enhance affordability and coverage for populations [3]. The World Health Organization (WHO) emphasises the pivotal role of traditional medicine in both disease prevention and treatment. It is not only recognised for its cost-effectiveness but is also noted as essential for achieving Universal Health Coverage (UHC) [4]. In low- and middle-income countries (LMICs), traditional medicine serves as an important resource for population health [5]. WHO statistics shows that 80% of people in Asian and African countries rely on various form of traditional medicine [6].

In the context of globalisation and the increasing emphasis on cooperative efforts, international collaboration among research institutions facilitates the exchange of knowledge and the generation of scientific evidence that transcends national borders, thereby advancing traditional medicine research and development (R&D) [7]. High-income countries (HICs) have a long-standing tradition of contributing to global R&D initiatives. Given the rising global demand for traditional medicine, fostering both the collaborative R&D efforts between HICs and LMICs and the South-South collaboration is essential. Such collaboration is crucial for the sustainable development of traditional medicine. Furthermore, it helps to decolonise global health by promoting traditional health innovation and practice in LMICs [8,9].

Traditional Chinese Medicine (TCM) has a rich history and has created a unique system of theories, diagnostics, and therapies. Within the diverse landscape of TCM, Chinese patent medicines (CPMs, referring to any TCMs formulated into a finished dosage form), stand out as a distinctive category that represent a significant blend of traditional and modern scientific principles [10,11]. It is among the most widely used traditional medical approaches worldwide. The collaborative R&D for CPMs allows global recognition for traditional medicines and the gap convergence between modern medicine and traditional medicine. China, in this way, possessed a distinctive engagement in the traditional medicine sustainable development by enabling R&D collaboration on CPMs [12]. Therefore, this study focuses on CPMs R&D as a means to contribute to the global effort in advancing traditional medicine.

Previous studies have mainly focused on analysing traditional medicine policies across various regions [13–16], and on exploring the usage and exportation of traditional medicine in different countries [17,18]. To the best of our knowledge, no empirical studies have been conducted to assess collaborative R&D and the contextual factors influencing traditional medicine. This study aims to provide a current overview of international collaborations on CPMs between China and other countries, focusing on quantitative R&D outputs of scientific research, patent applications, and clinical trials. It seeks to identify key contextual factors and strategies that can enhance R&D efforts in traditional medicine and support its sustainable development globally.

## METHODS

### Study design

This is a cross-sectional and descriptive study analysing international collaborative R&D outputs on Chinese patent medicines from 1996 to 2022.

### Data source and collection

#### International collaborative R&D output

We assessed the progress in international collaborative efforts for CPMs R&D across three dimensions of outputs: scientific research, patents, and clinical trials. The concept of international collaboration in this study is defined as joint participation in an article, patent, or clinical trial by researchers from both China and other countries. Data for scientific research, patent applications, and clinical trials were retrieved from the Web of Science (WOS), the Worldwide Patent Statistical Database (PATSTAT) [19], and the International Clinical Trials Registry Platform (ICTRP) [20], respectively. The Web of Science is an authoritative database of research publications and citations. The Worldwide Patent Statistical Database is a comprehensive databases containing bibliographical data related to more than 100 million patent documents from leading industrialised and developing countries. The Clinical Trials Registry Platform, established by the WHO, includes data from 17 national and regional clinical trial registries.

We used the keywords ‘Chinese traditional patent medicine’, ‘Traditional patent medicine’, ‘Chinese patent medicine’, ‘Chinese traditional patent formulation’, ‘Chinese patent formulation’, ‘Traditional patent drug’, ‘Traditional patent formulation’, or ‘Chinese herbal formulation’ in the search query. Specific search strategy was shown in Table S1 in the **Online Supplementary Document**. Items were excluded if the study, patent, or clinical trial was not explicitly designed for CPMs, such as those focusing on plant extracts, acupuncture, or massage. Additionally, exclusions were made if the article, patent, or clinical trial was not designed to evaluate treatment effect (*e.g*. those designed for toxicity tests) or did not have a specific treatment area. Over 11 428 outputs were identified using our search criteria during the review period. Of these, 1043 studies satisfied the eligibility criteria (Figure S1 in the **Online Supplementary Document**). Data on the date of publication, countries of collaborators, and the disease areas were extracted for the current analysis. The study period was from 1996 to 2022.

#### National multi-level characteristic

The collaborative countries were classified by income level based on the World Bank Country Group Classification into lower-income, low-middle-income, upper-middle-income and high-income countries [21]. We selected the following measures for each country:

1) gross domestic product (GDP) per capita (in thousand yuan);

2) health care access and quality (HAQ) index;

3) global innovation index (GII);

4) traditional medicine development score;

5) degree of cooperative institutionalisation;

6) globalisation index.

Gross domestic product per capita was obtained from the World Bank [22]; Healthcare access and quality index was obtained from Institute for Health Metrics and Evaluation (IHME) [23]; the global innovation index was acquired from the World Intellectual Property Organization (WIPO) [24]; and the globalisation index was obtained from the Swiss Economic Institute [25].

The traditional medicine development score was measured using an assignment method from 11 dimensions based on WHO Global Report on traditional and complementary medicine [26]. Each dimension was scored with a ‘Yes’ as 1 and a ‘No’ as 0. The dimensions include: national policy on TCM, national or state level laws or regulations on TCM, national office on TCM, national expert committee on TCM, national research institute for TCM, regulation of herbal medicines, registration system for herbal medicines, use of TCM, including indigenous medicine, provided by public sector, requirement for a license to practice TCM, inclusion of TCM in the national essential medicines list. The total score of each country ranges from 0 to 11. Based on their scores, countries are categorised into three development levels poor (0–3 points), moderate (4–7 points), and good (8–11 points). Internal consistency of the Traditional Medicine Development Score was tested using Cronbach’s alpha (α = 0.64), indicating acceptable reliability. The degree of cooperative institutionalisation was measured using an assignment method. Points were assigned to countries with a common collaborative mechanism and engagement in cooperation in traditional medicine, such as those in BRICS or the Shanghai Cooperation Organization. Each country was awarded one point for every year of collaboration, starting from the first year of their participation in such cooperative efforts. The cumulative points represent the degree of cooperative institutionalisation for each country. Test–retest reliability was evaluated using the intraclass correlation coefficient (ICC) (ICC = 0.83), suggesting good temporal consistency.

### Data analysis

Descriptive analyses were performed to summarise data from the outputs eligible for review. Outputs were categorised by the income group of collaborative countries, based on the World Bank classification [21]. Additionally, these outputs were categorised by the corresponding disease areas of focus, using the list of level-2 GBD causes acquired from the Institute for Health Metrics and Evaluation. The distribution and trends in retrieved outputs were assessed with respect to different collaborators’ income groups and disease areas.

The zero-inflated negative binomial regression (ZINB) model was used to investigate six sets of national factors associated with the number of collaborative R&D outputs. A ZINB model was chosen due to the presence of both over-dispersion and an excess of zero counts in the data. The ZINB model is a mixture model in which the outcome distribution consists of two parts [27]. Statistical analyses were performed using STATA, version 16 (StataCorp LLC, College Station, Texas, USA). All associations were presented as incidence rate ratios (IRR) or coefficients with corresponding 95% confidence intervals (Cis). Statistical significance was set at *P* < 0.05.

## RESULTS

### Collaborative R&D by countries

A total of 1043 R&D outputs were retrieved during the studying period, with the majority concentrated on scientific research (n = 613, 53.8%), followed by clinical trials (n = 337, 32.3%) and patent applications (n = 93, 8.9%). Most of the outputs involved collaboration with high-income countries (n = 964, 92.4%). The list of top ten active countries included eight high-income countries, one upper-middle-income country, and one lower-middle-income country, representing 84.6% of all collaborative outputs (Table S2 in the **Online Supplementary Document**). The USA participated in the highest percentage of the collaborative R&D (n = 518, 49.7%).

Low- and middle-income countries (LMICs) contributed only 7.6% of the total outputs (n = 79), with 21 countries involved. The most productive LMIC collaborators were Pakistan and Malaysia (each n = 14,), India (n = 9), Thailand (n = 6), and Brazil (n = 5), accounting for 60.8% of all LMICs outputs. Outputs from these collaborations were predominately in scientific research (n = 68), with fewer in clinical trials (n = 11) and none in patent application (n = 0) (**Table 1**).

**Table 1:** Number and percent of collaborative R&D outputs by country income groups

Country income group	High Income		Upper middle income		Lower middle income		Low income	
	**n**	**%**	**n**	**%**	**n**	**%**	**n**	**%**
Number of countries	28	57.1	7	14.3	11	22.4	3	6.1
Number of collaborative outputs								
Scientific research collaboration	545	88.9	27	4.4	34	5.5	7	1.1
Patent application collaboration	93	100.0	0	0	0	0	0	0
Clinical trials collaboration	326	96.7	5	1.8	6	1.5	0	0

**Figure 1:**
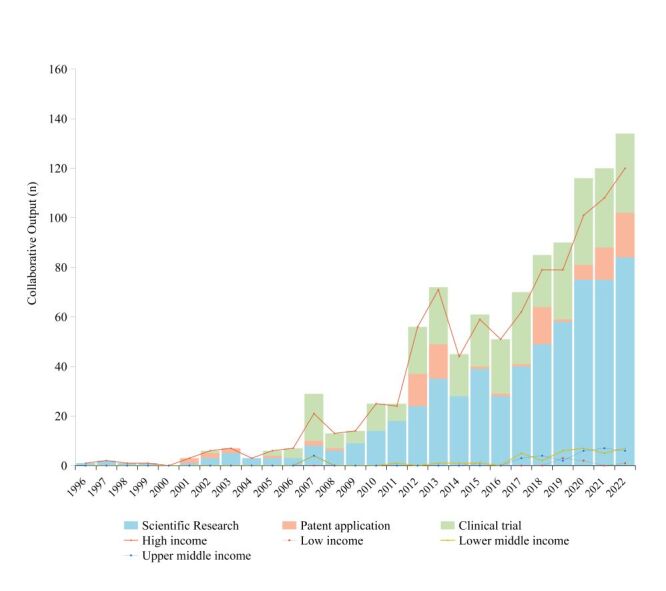
The trend of collaborative outputs by R&D phases and country income groups from 1996 to 2022.

Collaborative R&D outputs presented a consistent upward trend, rising from 1 in 1996 to 132 in 2022. This growth was observed across countries in various income groups (**Figure 1**). Despite the smaller absolute volume, LMIC collaborations increased at a compound annual growth rate of 30.42%, higher than the 19.40% of HICs. The proportion of outputs involving LMICs grew from 0% in 1996 to 11.7% in 2022.

### Subjected areas addressed in collaborative R&D output

Overall, collaborations predominately focused on non-communicable diseases (NCDs) R&D (n = 912, 87.4%), with cardiovascular disease accounting for the highest share (n = 151, 14.5%) (Figure S2 in the **Online Supplementary Document**). Communicable disease comprised only 12.6% (n = 131) of collaborations, with respiratory infections and tuberculosis being the most frequent (n = 110, 10.5% of the total collaborations). Other communicable diseases, including HIV/AIDS, sexually transmitted infections, enteric infections, neglected tropical diseases, and other infectious diseases, collectively contributed 2% of total R&D outputs.

**Figure 2:**
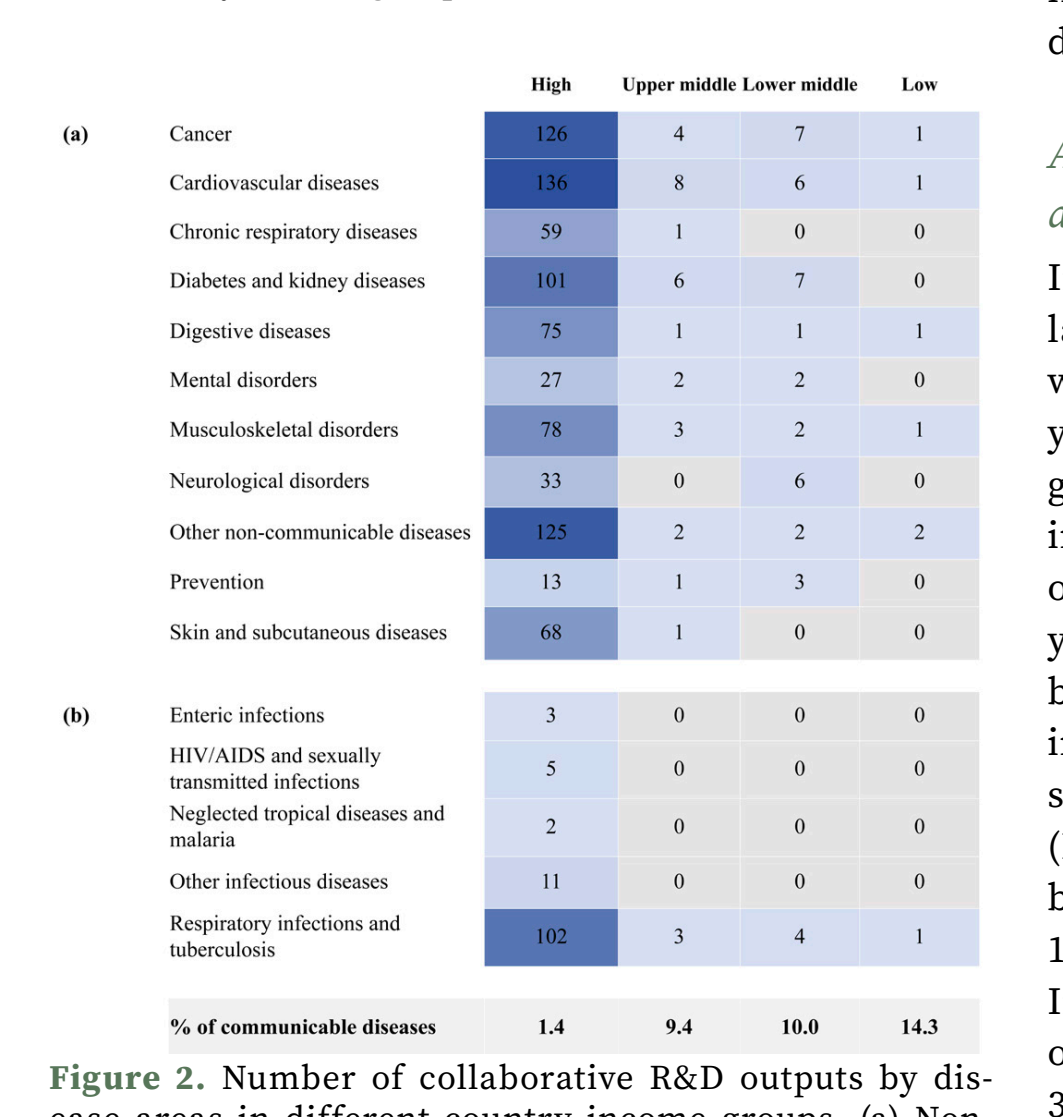
Number of collaborative R&D outputs by disease areas in different country income groups. (a) Non-communicable disease; (b) Communicable diseases.

Over time, the focus on communicable diseases has increased. In 1996, none of the collaborations targeted communicable diseases, whereas by 2022, this proportion rose to 28.8%. Differences were observed across income groups. Collaborations involving lower-income countries (LIMs) had a higher focus on communicable diseases (14.3%) than HICs (1.4%) (**Figure 2**). This suggests that disease priorities in collaborative R&D are shifting, with LMICs increasingly contributing to research on communicable diseases.

### Association between collaborative R&D output and countries’ characteristic

In the negative binomial regression modelling, collaborative outputs showed positive associations with the collaborator’s GDP-per-capital (in thousand yuan), traditional medicine development score, global innovation index, and degree of cooperative institutionalisation (**Table 2**). Specifically, for every one-unit increase in GDP-per-capita (in thousand yuan) in a collaborative country, outputs increased by 4% (IRR = 1.04; 95% CI = 1.03–1.06). Each one-unit increase in the degree of cooperative institutionalisation was associated with a 50% increase in outputs (IRR = 1.50; 95% CI = 1.13–1.99). Outputs increased by 131% (IRR = 2.31; 95% CI = 1.23–4.33) with every 1 standard deviation (SD) increase in the Global Innovation Index. Additionally, collaborative R&D outputs increased by 193% (IRR = 2.93; 95% CI = 2.22–3.85) with every 1 SD increase in the traditional medicine development score.

**Table 2:** The association between collaborative R&D output and countries’ characteristic

Factors	Country income group				IRR
	**High**	**Upper-middle**	**Lower-middle**	**Low**	
GDP per capital (thousand yuan)	50.62	9.66	3.24	0.85	1.04 (1.03–1.06)
HAQ index	91.57	64.41	50.51	35.14	1.00 (0.97–1.03)
Global innovation index	53.64	38.54	29.93	23.70	2.31 (1.23–4.33)
Traditional medicine development score	6.04	9.29	10.09	8.00	2.93 (2.22–3.85)
The degree of cooperative institutionalisation	1.25	2.00	1.64	1.33	1.50 (1.13–1.99)
Globalisation index	83.63	70.41	59.22	48.42	0.98 (0.94–1.01)

GDP – gross domestic product, HAQ – health care access and quality, IRR – incidence rate ratio

## DISCUSSION

Our study indicated that R&D collaboration on Chinese patent medicine in China remains largely centred on HICs, although collaboration with LMICs has shown an upward trend. Despite this increase, collaborative R&D on communicable diseases remains considerably limited compared to non-communicable diseases. LMICs’ contributions were more focused on communicable diseases compared to those from HICs. Collaborative research was associated with by partners’ higher economic and technological levels, the enhanced development of traditional medicine, and a higher degree of cooperative institutionalisation.

International collaborative R&D is known to increase research productivity and impact, promoting the science-based development of traditional medicine and accelerating its global access [28–31]. Our findings reflect improved inclusion of researchers from LMICs in traditional medicine R&D collaboration. The involvement of LMICs aids in global health decolonisation and aligns with studies highlighting the benefits of international research collaboration across various fields [32,33]. Recent evidence also indicates that contributions to R&D from LMICs are trending upward, which is critical for addressing health challenges [34–36]. However, despite important collaborative efforts by some countries, significant gaps remain in traditional medicine collaboration with LMICs compared to HICs, especially in those with more scientific activity, such as patent applications and clinical trials [37,38]. South-south collaboration on traditional medicine R&D is extremely important, given their extensive sources and experience with traditional medicines.

Results from our study indicated that collaboration on communicable diseases still lags notably behind, despite an increasing trend. Previous research has shown that traditional medicine has a curative effect on viral and bacterial infectious diseases, such as COVID-19 and malaria, offering unique advantages in improving clinical manifestation, inhibiting pathogens, and aiding organ recovery during severe and drug-resistant infections [39–42]. In many LMICs, traditional medicine is primarily used for infectious disease prevention and control, supported by strong historical evidence and public acceptance. Moreover, the application of traditional medicine in infectious diseases management has gained increasing significance, particularly after its practice during the COVID-19 pandemic [43]. To enhance the role of traditional medicine in achieving universal health coverage, it is essential to promote balanced R&D collaboration on communicable disease. This should address the most pressing needs and gaps in regions with higher disease prevalence but lower scientific activity.

Our findings extend existing knowledge by quantitatively identifying key contextual factors not previously empirically explored, such as institutional cooperation and traditional medicine development levels. Our findings align with existing literature suggesting that a myriad of factors, beyond economic and technological levels, influence scientific collaboration [44]. A solid cooperative foundation is crucial for promoting collaborative research. Declarations, plans, and programmes under cooperative mechanisms often address common concerns [45,46]. For example, the Joint Declaration of BRICS Countries on Strengthening Cooperation in Traditional Medicine focuses on innovation in traditional medicine products [47]. The Shanghai Cooperation Organization (SCO) has established alliances with the pharmaceutical industry, providing a platform for traditional medicine research [48]. Additionally, the development of traditional medicine helps promote collaborative research by bridging gaps in management, policy, regulation [49].

In addition to maintaining important collaborative ties with HICs like the USA and various European countries [39,40], LMICs have emerged as key hubs for international collaborations with China, especially in countries like Pakistan, Malaysia, and India. These countries have established a strong cooperative foundation and have made substantial progress in traditional medicine. Our analysis suggests effective strategies to foster global collaboration in traditional medicine R&D. Encouraging greater participation from LMICs with well-established traditional medicine practices and a solid foundation for collaboration could help drive global health decolonisation by advancing traditional and indigenous medicine practices in these regions. However, structural barriers, including limited funding, regulatory fragmentation, and language barriers, may restrict LMIC participation despite policy support [50]. Mechanisms such as regional R&D funds, shared trial registries, and policy harmonisation within ASEAN and BRICS frameworks could further promote sustainable South-South collaboration and balanced global participation in traditional medicine research.

To the best of our knowledge, this study is the first to map the landscape of collaborative R&D on traditional medicine. Moreover, this study comprehensively identifies contextual factors that influence collaboration, paving the way for enhanced collaboration in the field of Chinese patent medicine and other traditional medicines. However, there are some important limitations to note. First, our data sources were restricted to public databases, which limited the availability of data, despite our extensive use of keywords to retrieve relevant information. Second, our data, derived from scientific research, patent applications, and clinical trials, was used to explore the relationships between outcomes and independent factors. The limited number of participants in the patent application and clinical trial phases prevented us from providing phase-specific suggestions. Lastly, our analysis primarily focused on identifying associations between explanatory factors and outcomes, and thus, these associations should not be interpreted as causal relationships.

## CONCLUSIONS

Findings from this study underscored persistent gaps in the participation of low- and middle-income countries in collaborative R&D efforts for traditional medicine, especially when compared to high-income countries. The collaboration with low- and middle-income countries presented a more notable emphasis on communicable diseases. To promote the sustainable development of traditional medicine and enhance its contribution to global health goals, it is imperative to increase the involvement of low- and middle-income countries in global collaborations, especially south-south collaboration. This entails prioritising research agendas that align with local needs and focus on addressing communicable diseases effectively.

## Additional material


Online Supplementary Document
