# Supplementary material for: International collaborative research and development (R&D) on traditional medicine and its contextual factors: a cross-sectional analysis from 1996 to 2022
Source: J Glob Health. 2026 Feb 6;16:04029. doi: 10.7189/jogh.16.04029 (PMC12879262; doi:10.7189/jogh.16.04029)
Supplement: Online Supplementary Document [file jogh-16-04029-s001.pdf]

Supplement to: Sun YN, Ma JY, Dong JY, Gu YW, Lee MS, Ang L, Liu YM, Huang YM. International collaborative research and development (R&D) on traditional medicine and its contextual factors: A cross-sectional analysis from 1996 to 2022. J Glob Health. 2026;16:04029.

Table S1 Search strategy
Figure S1. Study flow diagram
Figure S2. Subjected areas addressed in collaborative R&D outputs
Table S2. Top ten countries that cooperated with China on CPMs R&D

Table S1 Search strategy

Database	Query	Result
Web of Science	TS=(Chinese Traditional Patent Medicine)) OR TS=(Traditional Patent Medicine)) OR TS=(Chinese Patent Medicine)) OR TS=(Chinese traditional patent formulation)) OR TS=(Chinese patent formulation)) OR TS=(traditional patent drugs)) OR TS=(traditional patent formulation)) OR TS=(Chinese herbal formulation)	6878
Worldwide Patent Statistical Database	Keyword(s) = “Chinese Traditional Patent Medicine” OR “Traditional Patent Medicine” OR “Chinese Patent Medicine” OR “Chinese traditional patent formulation” OR “Chinese patent formulation” OR “traditional patent drugs” OR “traditional patent formulation” OR “Chinese herbal formulation”	757
International Clinical Trials Registry Platform	“Chinese Traditional Patent Medicine” OR “Traditional Patent Medicine” OR “Chinese Patent Medicine” OR “Chinese traditional patent formulation” OR “Chinese patent formulation” OR “traditional patent drugs” OR “traditional patent formulation” OR “Chinese herbal formulation” in the Title OR “Chinese Traditional Patent Medicine” OR “Traditional Patent Medicine” OR “Chinese Patent Medicine” OR “Chinese traditional patent formulation” OR “Chinese patent formulation” OR “traditional patent drugs” OR “traditional patent formulation” OR “Chinese herbal formulation” in the Intervention	3793

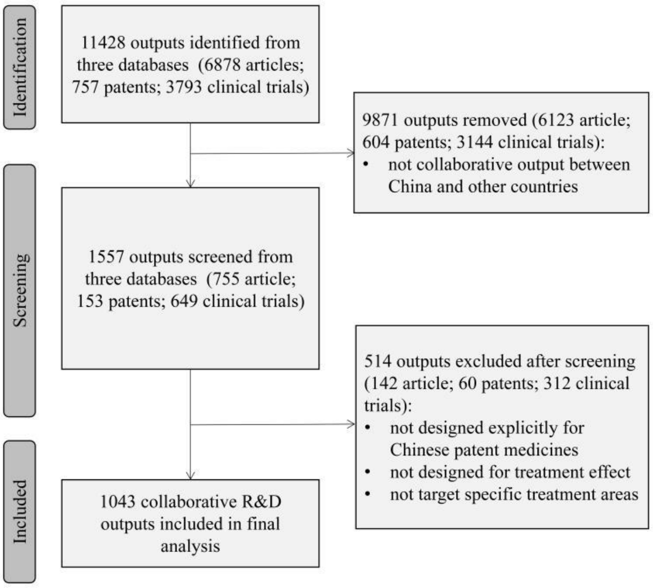

Figure S1. Study flow diagram

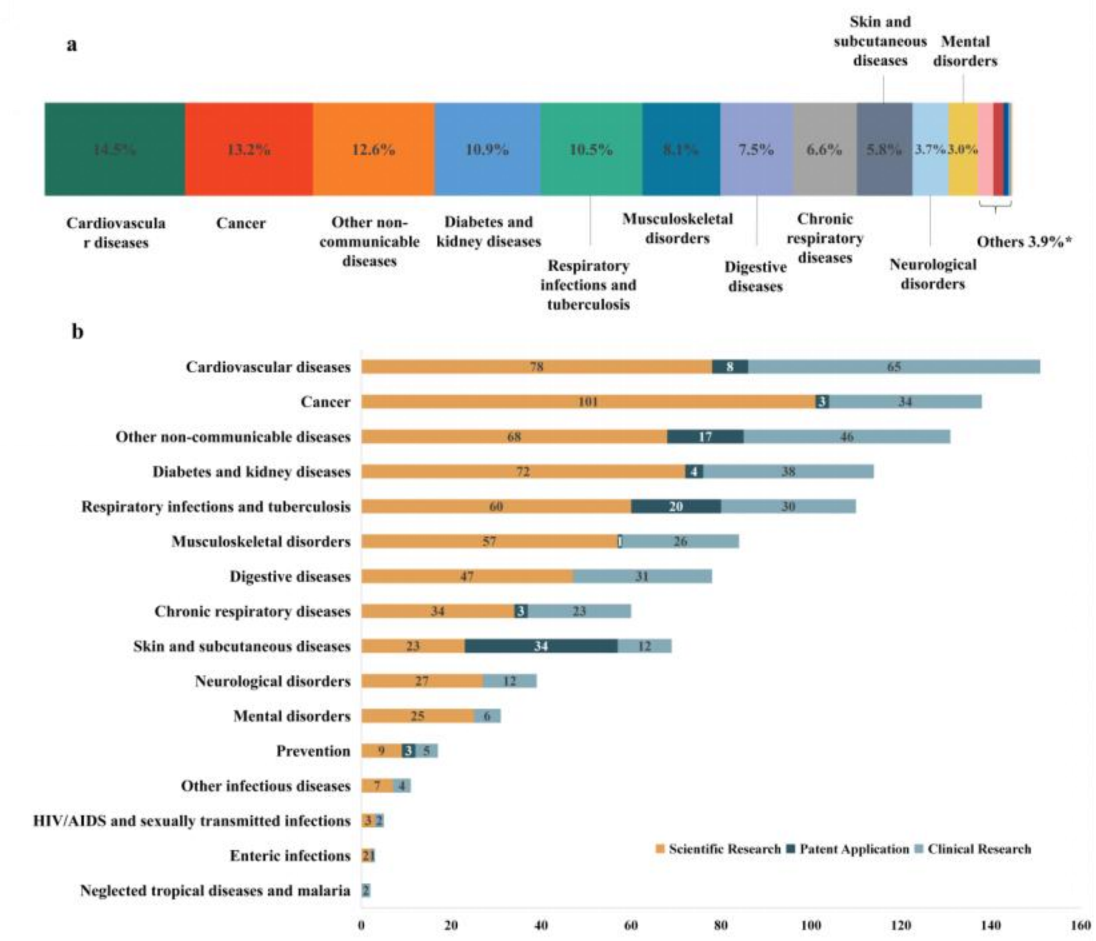

Figure S2. Subjected areas addressed in collaborative R&D outputs

**Others: Prevention 1.8%; Other infectious diseases 1.1%; HIV/AIDS and sexually transmitted infections 0.5%; Enteric infections 0.3%; Neglected tropical diseases and malaria 0.2%.*

Table S1. Top ten countries that cooperated with China on CPMs R&D

Country	Income Level	Total	Share	Average relative annual growth rate	Scientific research	Patent application	Clinical trial
United States of America	High	518	49.7%	224.8%	197	18	303
Australia	High	114	10.9%	54.8%	92	17	5
United Kingdom	High	81	7.8%	39.5%	63	4	14
Canada	High	44	4.2%	19.6%	40	3	1
Japan	High	43	4.1%	16.3%	41	2	0
Austria	High	22	2.1%	8.6%	18	0	4
Germany	High	20	1.9%	10.0%	15	0	5
Pakistan	Lower middle	14	1.3%	8.4%	12	0	2
Malaysia	High income	14	1.3%	7.6%	11	0	3
Italy	High	12	1.2%	5.6%	8	0	4
